# Sex Differences in the Role of Neurexin 3α in Zoster Associated Pain

**DOI:** 10.3389/fnint.2022.915797

**Published:** 2022-07-07

**Authors:** Phillip R. Kramer, Mikhail Umorin, Rebecca Hornung, M. Douglas Benson, Paul R. Kinchington

**Affiliations:** ^1^Department of Biomedical Sciences, Texas A&M University School of Dentistry, Dallas, TX, United States; ^2^Department of Ophthalmology and of Molecular Microbiology and Genetics, University of Pittsburgh, Pittsburgh, PA, United States

**Keywords:** orofacial, zoster, shingles, GABA, GABAergic, post-herpetic neuralgia, herpes zoster

## Abstract

Varicella zoster virus (VZV) induces orofacial pain and female rats show greater pain than male rats. During the proestrus phase of the estrous cycle the VZV induce pain response is attenuated in female rats. A screen of gene expression changes in diestrus and proestrus female rats indicated neurexin 3α (Nrxn3α) was elevated in the central amygdala of proestrus rats vs. diestrus rats. GABAergic neurons descend from the central amygdala to the lateral parabrachial region and Nrxn3α is important for presynaptic γ-Aminobutyric acid (GABA) release. Thus, we hypothesized that the reduced orofacial pain in male rats and proestrus female rats is the result of increased Nrxn3α within the central amygdala that increases GABA release from axon terminals within the parabrachial and inhibits ascending pain signals. To test this hypothesis Nrxn3 α expression was knocked-down by infusing shRNA constructs in the central amygdala. Then GABA release in the parabrachial was quantitated concomitant with measuring the pain response. Results revealed that knockdown of Nrxn3α expression significantly increases the pain response in both male rats and proestrus female rats vs. diestrus rats. GABA release was significantly reduced in the parabrachial of male and proestrus female rats after Nrxn3α knockdown. Neuronal activity of excitatory neurons was significantly inhibited in the parabrachial after Nrxn3α knockdown. These results are consistent with the idea that Nrxn3 within the central amygdala controls VZV associated pain by regulating GABA release in the lateral parabrachial that then modulates ascending orofacial pain signals.

## Introduction

Orofacial pain is often higher in females vs. males and this pain varies over the menstrual cycle (Falls et al., 1985; Suenaga et al., 2001; LeResche et al., 2003). Our lab used a varicella zoster virus associated (VZV) pain model because VZV induces herpes zoster within the body including the face (Ragozzino et al., 1982; Pavan-Langston, 1995; Pevenstein et al., 1999). This animal pain model closely correlates to humans having herpes zoster and post-herpetic neuralgia pain (Fleetwood-Walker et al., 1999; Kennedy et al., 2001; Dalziel et al., 2004; Hasnie et al., 2007; Guedon et al., 2015). In female rats orofacial pain, including varicella zoster virus (VZV) induced orofacial pain, differs during the estrous cycle (Flake et al., 2005; Kerins et al., 2005; Dong et al., 2007; Tashiro et al., 2007, 2008; Kramer and Bellinger, 2009; Stinson et al., 2017). The pain response is due, in part, to expression of early viral proteins (Guedon et al., 2014) and it has been shown that complete replication and production of new virus particles is not required for the pain response (Warner et al., 2021). Recently our lab reported neurexin 3α (Nrxn3α) was expressed in the central amygdala and parabrachial and that expression differed during the estrous cycle in rats with VZV induced pain (Hornung et al., 2020). Nrxn3α expression was elevated in the central amygdala of proestrus female rats vs. rats in diestrus.

The parabrachial is known to control orofacial pain signals (Rodriguez et al., 2017; Raver et al., 2020) and the amygdala controls affective orofacial pain (Nascimento et al., 2020; Askari-Zahabi et al., 2022). Neurexin 3α (Nrxn3α) is important for presynaptic γ-Aminobutyric acid (GABA) release (Aoto et al., 2015) and parabrachial GABA release inhibits neuronal signals ascending from the trigeminal nucleus and trigeminal ganglia (Rodriguez et al., 2017; Raver et al., 2020). Most neurons in the central amygdala are inhibitory GABAergic cells (Duvarci and Pare, 2014; Raver et al., 2020). Moreover, GABAergic neurons within the central amygdala regulate pain by inhibiting activity within the lateral parabrachial (Raver et al., 2020). From this established data we hypothesized that the reduced orofacial pain in male rats and proestrus female rats is the result of increased Nrxn3α within the central amygdala that increases GABA release from axon terminals within the parabrachial and inhibits ascending pain neurons.

In this study the expression of Nrxn3 α within the amygdala was attenuated in both male and cycling female rats. The effect on parabrachial neuronal activity and the amount of GABA release in the parabrachial were quantitated during behavioral pain testing.

## Materials and Methods

### Animal Husbandry

This study was carried out in accordance with the recommendations of Institutional Animal Care and Use Committee Guidebook and Texas A&M University College of Dentistry Institutional Animal Care and Use Committee. The animal protocol was approved by the Texas A&M University College of Dentistry Institutional Animal Care and Use Committee. Transgenic rats [Rat Resource and Research Center strain LE-Tg (Gad1-iCre) 3Ottc, RRRC#: 00751, developed by Brandon Harvey and Jim Pickel] were kept on a 14:10 light/dark cycle. The rats were given food and water *ad libitum*.

### Treatment and Experimental Groups

To knockdown Nrxn3α expression in the central amygdala this region was infused bilaterally with a lentivirus containing an shRNA expression construct. 4 weeks after infusion of the amygdala the vibrissae pads were injected with VZV or no VZV. 2 weeks after VZV injection animals were sacrificed and the central amygdala was isolated for ELISA analysis.

To measure GABA release and the pain response the central amygdala was infused bilaterally with a lentivirus containing a shRNA expression construct and the right lateral parabrachial was infused with a virus expressing an engineered fluorescent protein for measuring post synaptic GABA release termed iGABASnFR (Marvin et al., 2019). An optical fiber was placed in the right lateral parabrachial to measure fluorescence due to GABA binding this engineered protein. 4 weeks after infusion and lens placement VZV or control was injected into the left vibrissae pad. 2 weeks after VZV injection the effect of shRNA on parabrachial GABA release was quantitated during behavioral pain testing. After testing the animals were sacrificed and cell counts of c-fos positive cells that colocalized with the excitatory marker VGLUT2 were quantitated in the lateral parabrachial.

### Infusion and Lens Placement

Rats (280 g) were anesthetized with 2% isoflurane and an air flow of 2 L per minute. Using sterile technique a Hamilton infusion needle (Neuros #7002) was inserted into the brain. The amygdala was infused bilaterally with 1.0 μl of 1 × 10^7^ TU/ml lentivirus (pGFP-C-shLenti Nrxn3 shRNA, Catalog #TL712174V, or scrambled shRNA control lentivirus Catalog #TR30021V, Origene, Rockville MD). The rats were infused at coordinates anterior-posterior = 2.2 mm from Bregma, midline 4.2 mm and depth 8.4 mm, flat skull. In this same surgery the right lateral parabrachial was infused (in a portion of the rats) with 1 μl AAV1 pAAV.hSynap.iGABASnFR (Addgene) at stereotaxic coordinates anterior-posterior = 0.0 mm from Lambda, 2.4 mm from midline and at a depth of 6.6 mm, flat skull. A Stoelting stereotaxic syringe pump system was used to infuse at a rate of 50 nl per minute. After infusion the needle was left in place for 5 min and then removed. A single, clear borosilicate glass lens 9.0 mm length by 0.43 mm wide made by Doric Lenses (Quebec Canada, MFC_400/430-0.66_9mm_MF1.25_FLT) was immediately placed on the right side at coordinates anterior-posterior = 0.0 mm from Lambda, midline = 2.4 mm and depth = 6.5 mm. The lens was held in place with four stainless steel screws placed within the skull and dental cement (Metabond, Parkell Inc., Edgewood, NY).

### Varicella Zoster Virus Treatment

4 weeks after virus infusion the left vibrissae pad(s) was injected with 100 μl of MeWo cells infected with VZV (50,000 pfu/μl). Animal behavior was tested 2 weeks after VZV injection as this dose of VZV results in the greatest pain response (Kramer et al., 2017). MeWO cells are a human skin cell line in which VZV can replicate but cell free VZV is very unstable in the environment thus, VZV infection is completed by injecting MeWo cells containing virus. No VZV control groups were injected with 100 μl of MeWo cells containing no virus. Following behavioral testing the animals were sacrificed and tissue collected for molecular studies.

### Fiber Photometry Procedures

iGABASnFR is an engineered receptor that fluoresces upon binding GABA for the purpose of quantitating GABA release (Marvin et al., 2019). iGABASnFR is driven by the human synapsin gene for targeting expression in neurons. An optical fiber was placed in the parabrachial to measure this fluorescent signal after virus infusion. Two weeks after VZV injection fluorescent activity was measured using the RZ10X instrument and Synapse software (Tucker-Davis Technologies, Alachua, FL) during behavioral testing. During fluorescent measurement the parabrachial was excited at 465 nm using an LED light. Simultaneously a 405 nm LED light was used as an isobesic fluorescent signal to measure motion artifacts. The ΔF/F of the two signals was calculated using software developed in Python and the area under the curve for the positive ΔF/F peaks were analyzed with Prism 7.05 (GraphPad Software, La Jolla, CA). Positive peaks less than 10% of the baseline signal were excluded.

### Fluorescence Data Analysis

The neuronal signal was calculated using a least-squares regression line between the 465 nm fluorescence signal (dependent variable) and the 405 nm fluorescence signal (independent variable) (Lerner et al., 2015). Residuals resulting from prediction of the 465 nm signal using the 405 nm signal were divided by the predicted 465 nm signal giving the ΔF/F. Effectively, these are regression residuals normalized by corresponding the estimated values. Since regression tends to minimize any deviations from the estimated line, values within the −0.5 to 0.5 s peri-stimulus window were excluded from line-fitting to avoid reduction of potential ΔF/F signal due to stimulus. Pre- (−0.5 to 0.0 s) and post-stimulus (0.0–0.5 s) area under the curve (AUC) was calculated from ΔF/F time series values. Their difference were used as an effect in treatment comparisons.

### Behavioral Testing

Place Escape/Avoidance Paradigm (PEAP) testing was performed during the morning of the light phase to determine pain. To accomplish this, the rats were placed in a 30 cm × 30 cm × 30 cm acrylic box. The box has four walls and floor with the top of the box open and half the box is covered in black cloth on the outside of the acrylic. This test chamber was modeled from the PEAP test performed by the Fuchs’s laboratory (LaBuda and Fuchs, 2000). This assay was used to measure the motivation/affective aspect of pain (LaBuda and Fuchs, 2000; Baastrup et al., 2011). The PEAP test is based on the assumption that if animals escape and/or avoid a noxious stimulus, then the stimulus is aversive to the animal. Rodents being nocturnal in nature preferred to stay on the dark side when placed into the test chamber. After placing the rat in the test chamber, the rat was immediately poked with a 60-g filament every 15 s on the injected side if the rat was on the dark side and on the non-injected side if it was on the light side. A poke on the left hand side was marked by a button press and the timestamp of the button press would identify the fluorescent or electrical signal at that moment. Because VZV was injected into the vibrissae pad the target region for the poking was the area below the eye and caudal to the vibrissae pad. This region is innervated by the second branch of trigeminal ganglion (DaSilva and DosSantos, 2012), the nerve infected by VZV injection of the vibrissae pad. The time spent on the dark side of the box was recorded in 5 min bins and testing was performed for a total of 30 min. Thus, the theory behind the test is that if the rat is experiencing VZV induced pain when poked in the sensitive area it will not stay on it preferred dark side but will move to the non-preferred light side and stay there to avoid the poke.

### Vaginal Smears

Each female rat’s vagina was lavaged twice a day at 0,800 and 1,500 h using 250 μl of sterile 0.9% saline, and the solution was then transferred to a glass slide (StatLab, Inc., Lewisville, TX). The slides were completely dried and then fixed and stained using a Hema-Diff rapid differential stain kit (Anapath; StatLab). After staining, the cell morphologies were observed with a microscope and recorded.

### Immuno-Fluorescent Staining

A subset of animals were sacrificed by injecting with 100 mg/kg ketamine and 10 mg/kg xylazine. After injection the animals were perfused with 9% sucrose followed by 4% paraformaldehyde in PBS pH 7.4. Fixed tissues were stored in 25% sucrose, frozen, cryo-sectioned and the 32 μm sections placed on Histobond slides (VWR international, Radnor, PA). The tissue was post-fixed for 5 min in 4% paraformaldehyde, rinsed and then blocked for 2 h at room temperature with a PBS solution containing 5% normal goat serum (Sigma-Aldrich, St. Louis, MO) and 0.3% Triton-X 100. The slides were then incubated in a primary antibody solution overnight at 4°C. The primary antibody consisted of a mixture of the rabbit c-fos antibody (Millipore catalog # PC05) diluted to 2 μg/ml and mouse VGLUT2 antibody (Millipore catalog # MAB5504) at a 1:300 dilution. The primary antibodies were diluted with PBS, 5% BSA and 0.3% Triton X-100. After incubation in primary antibody the slides were then rinsed three times in PBS and 0.3% Triton-X 100 for a total of 45 min and placed for 2 h in secondary antibody and PBS and 0.3% Triton X-100. Secondary antibodies (1:500 dilution) included a mixture of goat anti-mouse 647 and goat anti-rabbit 568 (Invitrogen, Carlsbad, CA). After rinsing the slides three times in PBS and 0.3% Triton X-100 for a total of 45 min, the slides were treated with TrueVIEW Autofluorescence quenching kit (Vector Labs, Burlingame, CA), mounted with Fluoromount-G mounting medium containing Hoechst 33342 stain (Electron Microscopy Sciences, Hatfield, PA). The fluorescent signal was imaged using a Nikon fluorescent microscope, NIS-Elements imaging software and a Photometrics CoolSnap K4 CCD camera (Roper Scientific, Inc., Duluth, GA) or a Leica Stellaris 8 confocal microscope (Leica Microsystems, Wetzlar, Germany). Controls eliminating the primary antibody showed no signal (data not shown). Animals that did not have adequate perfusion, resulting in poor identification of cells after immunostaining, were eliminated from the counts.

Cell counts were completed by a reviewer blinded to the identity of the treatment groups. Every other section was selected for staining. Typically three sections were counted for each animal. The slides were analyzed using Image J software, the average background for the slides within a treatment group was subtracted from the image and a fluorescent signal associated with a cell nucleus was counted as a positive cell. Counts were completed for the number of c-fos/VGLUT2 stained cells within a 0.125 mm^2^ field. Counts were completed within the lateral parabrachial nucleus in a region that was infused with GABASnFR viral construct. Cell counts from two fields on each section were then averaged. This average count for the three sections was averaged for each animal. Values were given as a mean and standard error of the mean (SEM) for the animals in each treatment group.

### Enzyme-Linked Immunosorbent Assay

A separate group of animals infused with shRNA were sacrificed by exposure to CO_2_ and the brain was isolated after decapitation. The brain was placed on dry ice and sectioned into 2 mm slices (Zivic Instruments, Pittsburg, PA). From these slices a 2 mm circular punch of the central amygdala was placed in 250 μl of T-Per tissue protein extraction reagent containing Halt Protease Inhibitor and ground (Thermo Scientific, Rockford, IL). Ground samples were frozen and thawed, followed by centrifugation and decanting of the supernatant. Quantitation of vesicular Nrxn3α in the supernatant was completed on duplicate 100 μl samples of supernatant using an Nrxn3α enzyme-linked immunosorbent assay (ELISA) following the manufacturer’s directions (MyBiosource, San Diego, CA). Total protein was determined in each sample using a BCA protein assay (Thermo Scientific, Waltham, WA). Values represent the pg of Nrxn3α per μg of total protein.

### Statistics

PEAP and iGABASnFR data was normal (*p* > 0.05) in the Shapiro-Wilk normality test. Protein expression data was normal in the D’Agostino and Pearson normality test. PEAP data was analyzed by two-way ANOVA with a repeated measure of time. PEAP data was analyzed with repeated measures because data was collected multiple times over a 30 min period from each rat. The independent variable was the time spent on the light or dark side of the box (collected in 6 time bins i.e., 5, 10, 15, 20, 25, and 30 min) and the dependent variable was treatment (i.e., shRNA and sex/estrous cycle). When a significant effect was observed a Sidak’s multiple comparison test was completed (Prizm 7.05, GraphPad Software, La Jolla, CA). iGABASnFR and protein expression data was analyzed using two-way ANOVA and the independent variable was fluorescent signal or protein content and the dependent variable was treatment (i.e., shRNA and sex/estrous cycle). When a significant effect was observed a Tukey’s multiple comparison test was completed (Prizm 7.05). Cell count data was analyzed with the non-parametric Mann-Whitney test.

## Results

### Nrnx3α Expression Was Reduced in the Central Amygdala After Treatment With Nrxn3 shRNA

**FIGURE 1:**
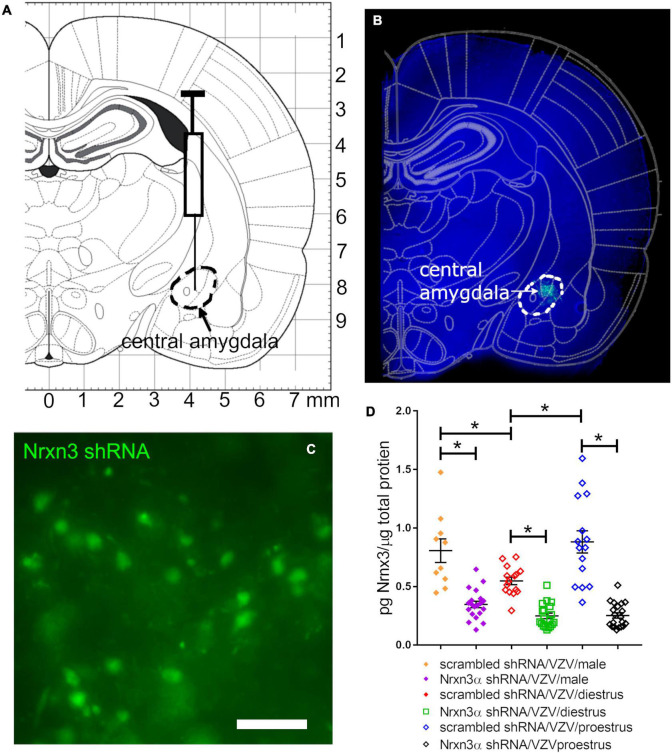
Lentivirus was injected in the central amygdala of Long Evans rats and produced NRXN3α shRNA that attenuated expression. **(A)** Shows an atlas image for infusion of the central amygdala indicated by a dashed black line. **(B)** Shows a 30 mm thick section from a representative female rat having GFP expression (green) within the central amygdala 6 weeks after infusion of lentivirus containing pGFP-C-shLenti Nrxn3 shRNA. **(C)** Is a high magnification image of GFP positive cells within the central amygdala. Bar = 50 μm. **(D)** Is a histogram of Nrxn3α protein expression in the right central amygdala using ELISA analysis. Nrxn3α expression was quantitated after infusion of either scrambled (control) shRNA or Nrxn3α shRNA and injection of VZV in the left vibrissae pad. Each point on the histogram represents an individual animal and the asterisk indicates *p* < 0.05.

Lentivirus was infused into the central amygdala (Figure 1A) resulting in expression of the shRNA construct (Figure 1B, green). shRNA was present in cells with a neuronal shape (Figure 1C). Nrnx3 α shRNA treatment significantly reduced Nrnx3α protein levels in comparison to rats treated with control shRNA that had a scrambled sequence *F*(1, 93) = 125, *p* < 0.0001. Nrnx3α knockdown was observed in both male and female rats (Figure 1D) and there was a significant effect of sex and estrous cycle on Nrnx3α protein content within the central amygdala *F*(2, 93) = 8, *p* < 0.0006. A significant interaction between sex/estrous cycle and shRNA treatment was observed *F*(2, 93) = 5.7, *p* < 0.004. When comparing proestrus rats to diestrus rats the proestrus rats had a significantly greater amount of Nrnx3α expression (Figure 1D). Male rats also had a significantly greater level of Nrnx3α expression vs. diestrus rats (Figure 1D). Diestrus and proestrus rats injected with control MeWo cells (no VZV group) had significantly (*p* < 0.05) less Nrnx3α expression than rats injected with VZV (compare no VZV/diestrus (0.49 ± 0.024, *n* = 6) to the scrambled/VZV/diestrus group (Figure 1D) and no VZV/proestrus (0.49 ± 0.026, *n* = 6) to the scrambled/VZV/proestrus groups (Figure 1D).

### Knocking Down Nrnx3α Increased the Varicella Zoster Virus Associated Pain Response

**FIGURE 2:**
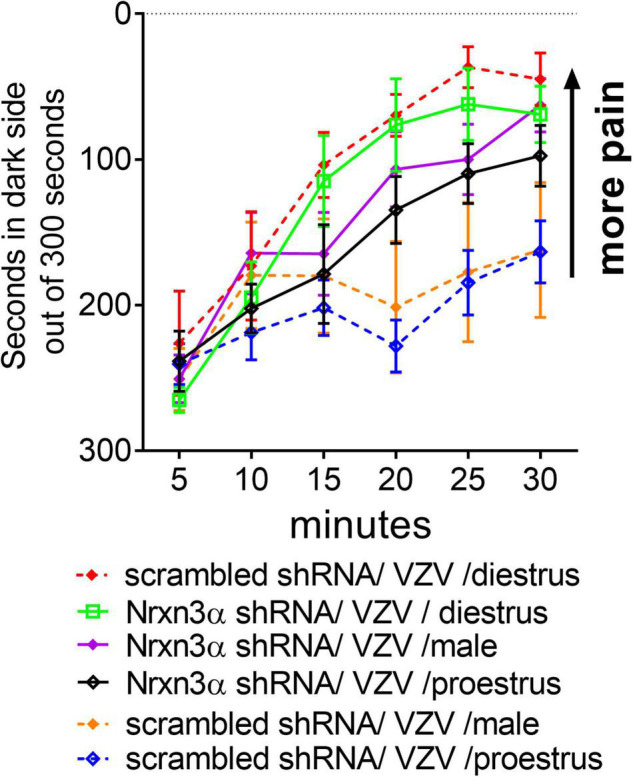
Attenuating Nrxn3α expression in the central amygdala increased VZV associated pain. The central amygdala of male and female rats were bilaterally infused with lentivirus expressing either scrambled shRNA (control shRNA) or Nrxn3α shRNA. The right lateral parabrachial was infused with AAV1 containing an engineered GABA receptor (iGABASnFR) and a permanent optical fiber was implanted in the right lateral parabrachial. Four weeks after brain surgery the left vibrissae pad was injected with VZV. 2 weeks after VZV injection pain behavior was measured in males and at specific estrous cycle phases in females.

Treatment with Nrnx3α shRNA increased the VZV associated orofacial pain response as compared to rats treated with scrambled (i.e., control) shRNA *F*(5, 44) = 4.9, *p* < 0.001. A significant interaction between time and shRNA treatment was observed in the animals injected with VZV *F*(25, 225) = 2.1, *p* < 0.001. In male rats, administering Nrnx3 shRNA increased the VZV associated orofacial pain response as compared to rats treated with scrambled shRNA *F*(1, 21) = 6.2, *p* = 0.02 (compare Nrnx3α shRNA/VZV/male group to scrambled shRNA/VZV/male group, Figure 2). Rats in proestrus had a significantly greater pain response after Nrnx3α shRNA treatment *F*(1, 12) = 7.8, *p* = 0.015 (compare Nrnx3α shRNA/VZV/proestrus group to scrambled shRNA/VZV/proestrus group, Figure 2). Nrnx3α knockdown did not significantly affect the pain response in diestrus rats injected with VZV (compare Nrnx3α shRNA/VZV/diestrus group to scrambled shRNA/VZV/diestrus group, Figure 2). The no VZV control showed no significant pain response (data not shown).

### GABA Release Is Inhibited by Knocking Down Nrnx3α Expression

**FIGURE 3:**
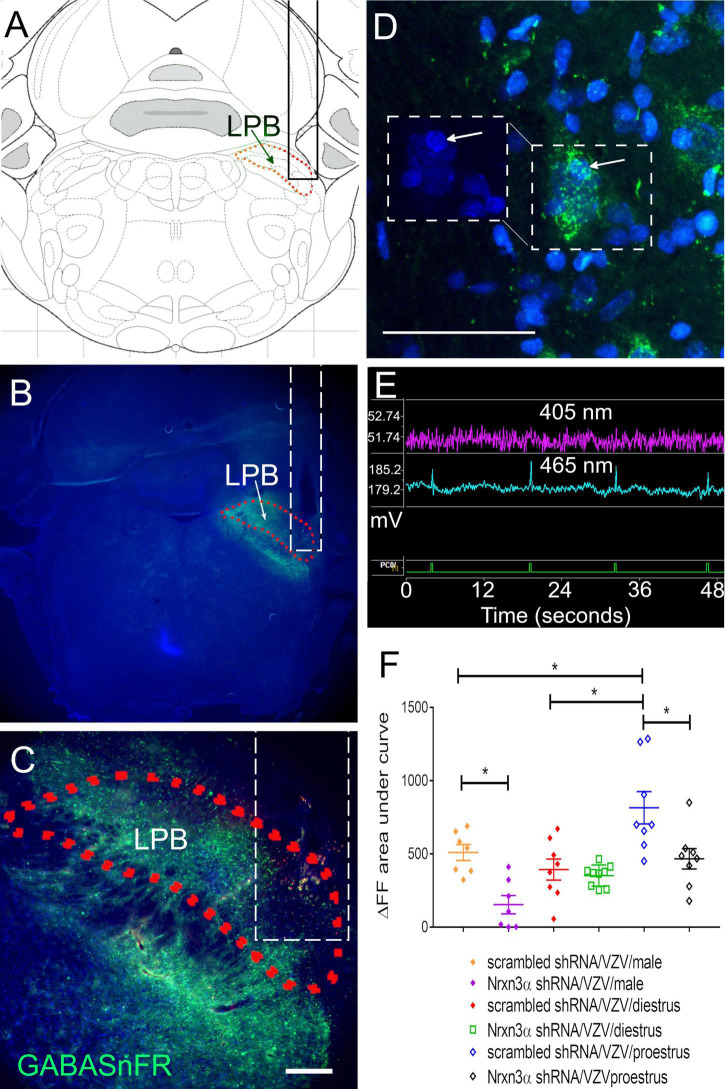
Fluorescent image of lateral parabrachial from Long Evans rats after infusion of the parabrachial with an iGABASnFR construct. The central amygdala of male rats was bilaterally infused with lentivirus expressing either scrambled shRNA virus (control shRNA) or Nrxn3α shRNA. The right lateral parabrachial (LPB) was infused with AAV1 containing an engineered GABA receptor (iGABASnFR). **(A)** Permanent optical fiber was implanted in the right lateral parabrachial after infusion of AAV. The outline of the optical fiber is shown as a solid black in **(A)** or dashed white line in **(B,C)**. Four weeks after brain infusion surgery the left vibrissae pad was injected with VZV and the animals sacrificed 2 weeks after injection for isolation of the brain. **(A)** Shows a brain atlas image corresponding to the section in **(B)**. **(B)** Is a low magnification image of the iGABASnFR fluorescent signal (green) is shown within the lateral parabrachial nucleus (outlined with a red dotted line) from a representative male rat. In **(C)**, the same parabrachial region is outlined by a red dotted line and is magnified. Bar = 200 μm. In **(D)**, several magnified cells within the lateral parabrachial region stain for iGABASnFR (green). Boxed region on the right shows iGABASnFR (green) and boxed region on the left shows Hoechst 33342 nuclear stain (blue), arrow points to the same cell. Bar = 50 μm. **(E)** Shows the isobesic 405 nm fluorescent signal (purple) measured in millivolts (y-axis, mV) over time (seconds, x-axis). The 405 nm fluorescent signal is related to motion artifacts. The electrical signal generated from the 465 nm fluorescent iGABASnFR signal (blue trace) results from excitation of iGABASnFR. Data was collected using the Tucker-Davis Instrument and Synapse software. The ΔF/F was calculated using the 405 nm and 465 nm signals. The green trace at the bottom is when the button was pressed (PCO/is the button device connection slot into the instrument). A spike in the green trace indicates a button press. The animal was poked at the same time a button was pressed during behavioral testing. **(F)** Shows the ΔF/F fluorescent signal for the engineered receptor resulting from binding GABA. Each point on the histogram represents an individual animal and the asterisk indicates *p* < 0.05.

Measurement of GABA release within the lateral parabrachial was completed by infusion of the parabrachial (Figure 3A) with iGABASnFR producing AAV (Figures 3B–D). Individual cells express the engineered GABA receptor (i.e., iGABASnFR) in a punctate manner (Figure 3D). Of the animals tested for pain, up to 2 animals per treatment group were eliminated from these GABA measurements because the histology indicated incorrect placement of the lens and there was no fluorescent signal. Fluorescent signal was captured during behavioral testing and a spike in the 465 nm fluorescent iGABASnFR signal (blue trace, Figure 3E) was observed after poking the rat whisker pad (spike in green trace indicates a poke, Figure 3E). GABA release was calculated using both the 405 nm and 465 nm signals (Figure 3E). Nrnx3α shRNA treatment significantly reduced iGABASnFR fluorescent signal in comparison to rats treated with scrambled shRNA *F*(1, 41) = 18.7, *p* < 0.0001. There was a significant effect of sex and estrous cycle on iGABASnFR fluorescence *F*(2, 41) = 11.4, *p* < 0.0005. A significant interaction between sex/estrous cycle and shRNA treatment was observed *F*(2, 41) = 3.4, *p* < 0.05. The amount of GABA released within the lateral parabrachial region was significantly reduced in male rats and proestrus female rats after Nrnx3α shRNA treatment vs. scrambled shRNA (Figure 3F). The proestrus female rats showed significantly more GABA release than diestrus rats and male rats (Figure 3F).

### Activity of Excitatory Neurons Increased in the Lateral Parabrachial After Nrnx3α Knockdown

**FIGURE 4:**
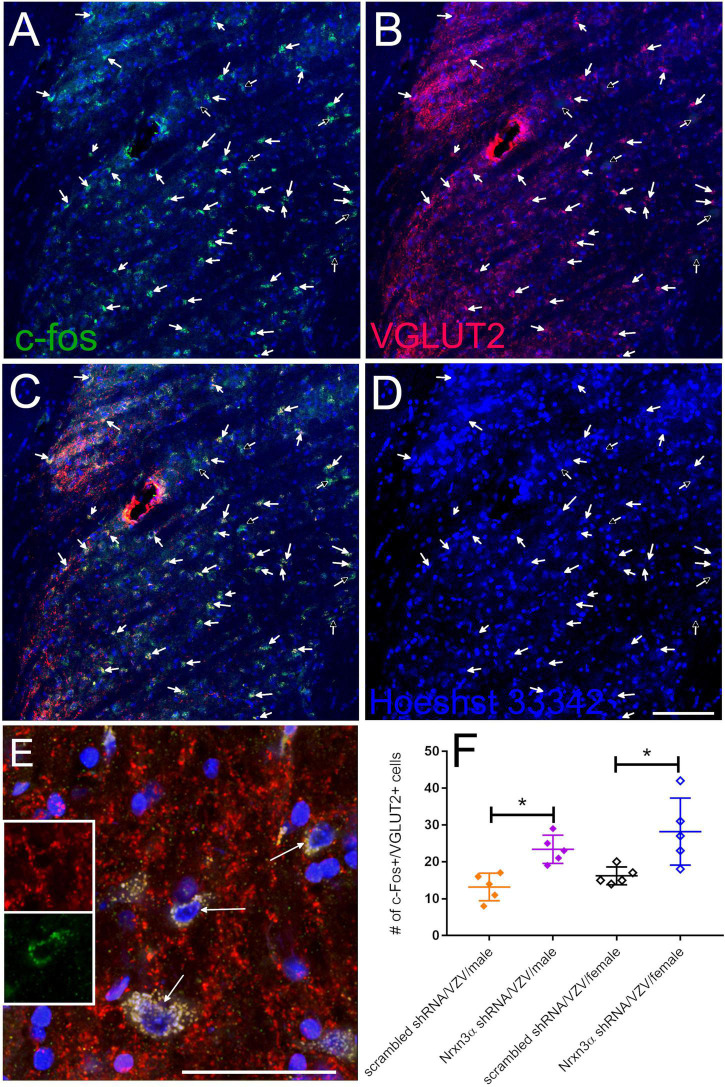
Excitable neurons had increased activity in the parabrachial after infusion of the central amygdala with Nrxn3α shRNA. Images are from the lateral parabrachial region of a female rat after infusion of the central amygdala with scrambled shRNA or Nrxn3α shRNA and after having injected the vibrissa pad with VZV. In **(A)**, the c-fos staining is shown in green and in **(B)** the VGLUT2 staining is shown in red. Cells that have c-fos and VGLUT2 colocalized are shown in yellow in **(C)**. Hoechst 33342 nuclear stain is blue in **(D)**. The arrows point to cells that were counted as c-fos and VGLUT2 positive. Open arrows points to cells that were considered c-fos positive but did not show a VGLUT2 signal. Bar = 100 μm. **(E)** Shows a high magnification of cells that have c-fos and VGLUT2 colocalized (yellow, arrows). Inserts show higher magnification image of the cell in the center, VGLUT2 (upper insert) and c-fos (lower insert). Bar = 50 μm. **(F)** Is a histogram of the counts from the different treatment groups for cells that had the c-fos and VGLUT2 fluorescent signal co-localized. Each point on the histogram represents an individual animal and the asterisk indicates *p* < 0.05.

c-fos expression is a marker for active neurons (Gao and Ji, 2009) and VGLUT2 is a marker for excitatory glutamatergic neurons (Varoqui et al., 2002). More VGLUT2 positive neurons colocalized with c-fos after Nrnx3α knockdown. c-fos positive neurons colocalizing with VGLUT2 were counted in the lateral parabrachial region after infusing the central amygdala with virus expressing Nrnx3α shRNA (Figures 4A–E, arrows) or a scrambled shRNA. Immunofluorescence indicated greater than 80% of the c-fos positive cells colocalized with VGLUT2 signal (Figures 4A–D compare arrows to open arrows). In Figure 4F a histogram indicates the number of c-fos positive cells colocalizing with VGLUT2 in both males and females. Nrnx3α knockdown significantly increased the number of c-fos positive cells colocalizing with VGLUT2 in both male and female rats. Vaginal smears were not performed before perfusion and the female rats have an unknown estrous cycle stage.

## Discussion

In female rats expression of Nrxn3α was elevated at proestrus in the central amygdala and the amount of transcript was correlated to the concentration of plasma estradiol (Hornung et al., 2020). In this study attenuating Nrxn3 expression within the central amygdala increased VZV associated orofacial pain after VZV was injected into the vibrissae pad of the rat. Male rats and proestrus female rats had significantly more Nrnx3α expression than diestrus female rats. Importantly, male rats and proestrus female rats had a reduced pain response as compared to diestrus female rats. Nrnx3α knockdown increased the pain response only in the male and proestrus rats. Attenuation of Nrxn3 expression in the amygdala also reduced GABA release in the male and proestrus rats but not in diestrus rats. Nrxn3 regulates GABA release from GABAergic neurons projecting from the amygdala and this release correlated to changes in pain. These results are consistent with the idea that Nrxn3 within the central amygdala controls VZV associated pain by regulating GABA release in the lateral parabrachial. Neuronal activity within excitable cells of the parabrachial was also elevated after Nrnx3α knockdown suggesting GABA modulates ascending orofacial pain signals.

Nrxn3α is important for presynaptic GABA release (Aoto et al., 2015) and because parabrachial GABA release inhibits neuronal signals ascending from the trigeminal nucleus and trigeminal ganglia (Rodriguez et al., 2017; Raver et al., 2020) it is likely that by reducing Nrxn3 expression within amygdala that ascending pain signals would be enhanced. Consistent with this idea the amygdala has been shown to control orofacial affective pain responses (Nascimento et al., 2020; Askari-Zahabi et al., 2022). Moreover, GABAergic neurons within the central amygdala can regulate pain by inhibiting activity within the lateral parabrachial (Raver et al., 2020).

VZV induced pain was enhanced in male and proestrus females due to Nrnx3α knockdown but proestrus female rats had significantly more GABA release than male rats (compare scrambled shRNA/VZV/proestrus group to the scrambled shRNA/VZV/male group). In contrast, the pain response was not significantly different between the scrambled shRNA/VZV/proestrus group and the scrambled shRNA/VZV/male group. Thus, the enhanced pain response after Nrnx3α knockdown was likely due, in part, by GABA release but may have also involved another mechanism. For example, Nrxn3 serves to suppress presynaptic release in females, but promote postsynaptic strength and synapse maintenance, including the number of synaptic connections, in males (Boxer et al., 2021). In the event that postsynaptic strength was decreased in males after Nrnx3α knockdown then the increased pain response was the result of both reduced GABA release and reduced postsynaptic strength. This idea could be tested by infusing the central amygdala with a synaptophysin conjugated fluorophore that would label the synaptic connections within the lateral parabrachial, the number of synaptic connections on specific cell types (e.g., excitatory cells) could be counted for each group (Boxer et al., 2021).

Sex differences were observed in that Nrnx3α knockdown increased the pain response in male rats but not in diestrus female rats. Moreover, proestrus female rats had a higher pain response than diestrus female rats after Nrnx3α knockdown consistent with the idea that sex steroids have a role in the how Nrnx3α modulates VZV pain. A mechanism explaining the sex differences is that sex steroids bind nuclear receptors that then act on the Nrnx3α promoter. Sex steroid estradiol was shown to modulate Nrxn3α expression (Aenlle and Foster, 2010). Nuclear estrogen receptors have been localized to the central amygdala (Shughrue et al., 1997; Shughrue and Merchenthaler, 2001). Estradiol likely alters the activity of neurons within the amygdala through estrogen receptors (Ueyama et al., 2006). Multiple potential estrogen response element sites are present in the first 1,500 bp of the Nrxn3α promoter. Alternatively, 30 progesterone receptor binding sites are present in the first 1,500 bp of the Nrxn3α promoter and could alter Nrnx3α expression.

In male and proestrus females Nrnx3α knockdown resulted in a decreased in GABA release but an increase in the pain response. Also, Nrxn3 knockdown attenuated excitatory activity in the lateral parabrachial of both male and female rats. These results are consistent with the idea that Nrnx3α within the central amygdala controls VZV associated pain by regulating GABA release in the lateral parabrachial through inhibition of excitatory neurons ascending from the orofacial region.

## Data Availability Statement

The raw data supporting the conclusions of this article will be made available by the authors, without undue reservation.

## Ethics Statement

The animal study was reviewed and approved by Texas A&M University School of Dentistry Institutional Animal Care and Use Committee.

## Author Contributions

PhK contributed to the planning, performing, data analysis, and writing of the manuscript. RH contributed to the planning and performing of the experiments. MU contributed to the data analysis and writing of the manuscript. MB contributed to the performing and data analysis of these experiments. PaK contributed to the planning and performing of the experiments.

## Conflict of Interest

The authors declare that the research was conducted in the absence of any commercial or financial relationships that could be construed as a potential conflict of interest.

## Publisher’s Note

All claims expressed in this article are solely those of the authors and do not necessarily represent those of their affiliated organizations, or those of the publisher, the editors and the reviewers. Any product that may be evaluated in this article, or claim that may be made by its manufacturer, is not guaranteed or endorsed by the publisher.
